# Sexual assault incidents among college undergraduates: Prevalence and factors associated with risk

**DOI:** 10.1371/journal.pone.0186471

**Published:** 2017-11-08

**Authors:** Claude A. Mellins, Kate Walsh, Aaron L. Sarvet, Melanie Wall, Louisa Gilbert, John S. Santelli, Martie Thompson, Patrick A. Wilson, Shamus Khan, Stephanie Benson, Karimata Bah, Kathy A. Kaufman, Leigh Reardon, Jennifer S. Hirsch

**Affiliations:** 1 Division of Gender, Sexuality and Health, Departments of Psychiatry and Sociomedical Sciences, New York State Psychiatric Institute and Columbia University Medical Center, New York, New York, United States of America; 2 Ferkauf Graduate School of Psychology, Yeshiva University, New York, New York, United States of America; 3 Department of Epidemiology, Mailman School of Public Health, Columbia University, New York, New York, United States of America; 4 Division of Biostatistics, Department of Psychiatry, New York State Psychiatric Institute and Columbia University Medical Center, New York, New York, United States of America; 5 Department of Biostatistics, Mailman School of Public Health, Columbia University, New York, New York, United States of America; 6 Social Intervention Group, School of Social Work, Columbia University, New York, New York, United States of America; 7 Heilbrunn Department of Population and Family Health, Mailman School of Public Health, Columbia University, New York, New York, United States of America; 8 Department of Youth, Family, and Community Studies, Clemson University, Clemson, South Carolina, United States of America; 9 Department of Sociomedical Sciences, Mailman School of Public Health, Columbia University, New York, New York, United States of America; 10 Department of Sociology, Columbia University, New York, New York, United States of America; University of West London, UNITED KINGDOM

## Abstract

Sexual assault on college campuses is a public health issue. However varying research methodologies (e.g., different sexual assault definitions, measures, assessment timeframes) and low response rates hamper efforts to define the scope of the problem. To illuminate the complexity of campus sexual assault, we collected survey data from a large population-based random sample of undergraduate students from Columbia University and Barnard College in New York City, using evidence based methods to maximize response rates and sample representativeness, and behaviorally specific measures of sexual assault to accurately capture victimization rates. This paper focuses on student experiences of different types of sexual assault victimization, as well as sociodemographic, social, and risk environment correlates. Descriptive statistics, chi-square tests, and logistic regression were used to estimate prevalences and test associations. Since college entry, 22% of students reported experiencing at least one incident of sexual assault (defined as sexualized touching, attempted penetration [oral, anal, vaginal, other], or completed penetration). Women and gender nonconforming students reported the highest rates (28% and 38%, respectively), although men also reported sexual assault (12.5%). Across types of assault and gender groups, incapacitation due to alcohol and drug use and/or other factors was the perpetration method reported most frequently (> 50%); physical force (particularly for completed penetration in women) and verbal coercion were also commonly reported. Factors associated with increased risk for sexual assault included non-heterosexual identity, difficulty paying for basic necessities, fraternity/sorority membership, participation in more casual sexual encounters (“hook ups”) vs. exclusive/monogamous or no sexual relationships, binge drinking, and experiencing sexual assault before college. High rates of re-victimization during college were reported across gender groups. Our study is consistent with prevalence findings previously reported. Variation in types of assault and methods of perpetration experienced across gender groups highlight the need to develop prevention strategies tailored to specific risk groups.

## Introduction

Recent estimates of sexual assault victimization among college students in the United States (US) are as high as 20–25% [1–3], prompting universities to enhance or develop policies and programs to prevent sexual assault. However, a 2016 review [4] highlights the variation in sexual assault prevalence estimates (1.8% to 34%) which likely can be attributed to methodological differences across studies, including varying sexual assault definitions, sampling methods, assessment timeframes, and target populations [4]. Such differences can hamper efforts to understand the scope of the problem. Moreover, while accurate estimates of prevalence are crucial for calling attention to the population-health burden of sexual assault, knowing more about risk factors is critical for determining resource allocation and developing effective programs and policies for prevention.

Reasons for the variation in prevalence estimates include different definitions of sexual assault and assessment methods. Under the rubric of sexual assault, researchers have investigated experiences ranging from sexual harassment at school or work, to unwanted touching, including fondling on the street or dance floor, to either unwanted/non-consensual attempts at oral, anal or vaginal sexual intercourse (attempted penetrative sex), or completed penetrative sex [3,5–7]. Some studies have focused on a composite variable of multiple forms of unwanted/non-consensual sexual contact [8,9] while others focus on a single behavior, such as completed rape [10]. Some studies focus on acts perpetrated by a single method (e.g. incapacitation due to alcohol and drug use or other factors) [11], while others include a range of methods (e.g., physical force, verbal coercion, and incapacitation) [12–15]. In general, studies that ask about a wide range of acts and use behaviorally specific questions about types of sexual assault and methods of perpetration have yielded more accurate estimates [16]. Behavioral specificity avoids the pitfall of participants using their own sexual assault definitions and does not require the respondent to identify as a victim or survivor, which may lead to underreporting [10,17–19].

Although an increasing number of studies have used behaviorally specific methods and examined prevalence and predictors of sexual assault [20,21], they typically have used convenience samples. Only a few published studies have used population-based surveys and achieved response rates sufficient to mitigate some of the concerns of sample response bias [4]. US federal agencies have urged universities to implement standardized “campus climate surveys” to assess the prevalence and reporting of sexual violence [22]. Although these surveys have emphasized behavioral specificity, many have yielded low response rates (e.g., 25%) [23], particularly among men [24], creating potential for response bias in the obtained data. Population-based probability samples with behavioral specificity, good response rates, sufficiently large samples to examine risk for specific subgroups (e.g., sexual minority students), and detailed information on personal, social, or contextual risk factors (e.g., alcohol use) [22,23] are needed to more accurately define prevalence and inform evidence-based sexual assault prevention programs.

Existing evidence suggests that most sexual assault incidents are perpetrated against women [25]; however, few studies have examined college men as survivors of assault [26–28]. Furthermore, our understanding of how sexual orientation and gender identity relate to risk for sexual assault is limited, despite indications that lesbian, gay, bisexual (LGB), and gender non-conforming (GNC) students are at high risk [29–31]. It is unclear if these groups are at higher risk for all types of sexual assault or if prevention programming should be tailored to address particular types of assault within these groups. Also, although women appear to be at highest risk for assault during freshman year [32,33], the dearth of studies with men or GNC students have limited conclusions about whether freshman year is also a risky period for them.

Additional factors associated with experiencing sexual assault in college students include being a racial/ethnic minority student (although there are mixed findings on race/ethnicity) [34,35], low financial status, and prior history of sexual assault [3,33,36]. Other risk factors include variables related to student social life, including being a freshman [24], participating in fraternities and sororities [19,37,38], binge drinking [1,39] and participating in “hook-up” culture [40–42]. Whether sexual assault is happening in the context of more casual, typically non-committal sexual relationships (“hook-ups”) [40] vs. steady intimate or monogamous relationships has important implications for prevention efforts.

To fill some of these knowledge gaps, we examined survey data collected from a large population-based random sample of undergraduate women, men, and GNC students at Columbia University (CU) and Barnard College (BC). The aims of this paper are to:

1. Estimate the prevalence of types of sexual assault incidents involving a) sexualized touching, b) attempted penetrative (oral, anal or vaginal) sex, and c) completed penetrative sex since starting at CU/BC;
2. Describe the methods of perpetration (e.g., incapacitation, physical force, verbal coercion) used; and
3. Examine associations between key sociodemographic, social and romantic/sexual relationship factors and different types of sexual assault victimization, and how these associations differ by gender.

## Materials and methods

This study used data from a population-representative survey that formed one component of the Sexual Health Initiative to Foster Transformation (SHIFT) study. SHIFT used mixed methods to examine risk and protective factors affecting sexual health and sexual violence among college undergraduates from two inter-related institutions, CU’s undergraduate schools (co-educational) and BC (women only), both located in New York City. SHIFT featured ethnographic research, the survey, and a daily diary study. Additionally, SHIFT focused on internal policy-translation work to inform institutionally-appropriate, multi-level approaches to prevention.

### Participants

Survey participants were selected via stratified random sampling from the March 2016 population of 9,616 CU/BC undergraduate students ages 18–29 years. We utilized evidence-based methods to enhance response rates and sample representativeness [22,43]. Using administrative records of enrolled students, 2,500 students (2,000 from CU and 500 from BC) were invited via email to participate in a web-based survey. Of these 2,500 students, 1,671 (67%) consented to participate (see Procedures). Among those who consented to participate, 80.5% were from CU and 19.5% were from BC (see Table 1 below for demographic data on the CU/BC student population, the random sample of students contacted, the survey responders, and the current analytic sample).

**Table 1:** Demographics of random sample and responders of undergraduates at CU/BC.

	Total undergraduate population (CU and BC)		Randomly selected sample of students		Responders—study participants who completed consent		Analytic sample—responded to sexual assault questions on survey		Cramer’s V^a^
	n	%	n	%	n	%	n	%	
**Total**	9616		2500		1671		1592		
**Gender**									
Female	5765	60%	1395	56%	956	58%	928	58%	0.030
Male	3851	40%	1105	44%	678	41%	634	40%	
GNC	NA	NA	NA	NA	26	2%	26	2%	
**Year in school**									
Freshman	2080	22%	533	21%	411	25%	396	25%	0.051
Sophomore	2287	24%	589	24%	404	24%	387	24%	
Junior	2483	26%	667	27%	435	26%	415	26%	
Senior^b^	2763	29%	711	28%	409	25%	391	25%	
**Age**									
18–20	5329	55%	1368	55%	911	55%	882	56%	0.041
21–23	3433	36%	879	35%	620	37%	587	37%	
24–29	854	9%	253	10%	130	8%	120	8%	
**Race/ethnicity**									
White Non- Hispanic	4159	43%	986	39%	708	44%	678	43%	0.086
Asian Non- Hispanic	2583	27%	637	25%	384	24%	359	23%	
Black Non- Hispanic	1046	11%	274	11%	137	9%	132	8%	
Hispanic	1104	11%	281	11%	246	14%	239	15%	
Other	724	8%	322	13%	154	10%	151	10%	
**US born**									
Yes	7925	82%	2053	82%	1251	76%	1203	76%	0.074
No	1691	18%	447	18%	394	24%	373	24%	
**Pell Grant**									
Yes	1694	18%	454	18%	362	23%	352	23%	0.057
No	7922	82%	2046	82%	1225	77%	1190	77%	

^a^Cramer’s V is a measure of effect size for the difference between the demographic distributions in the responders (n = 1671) vs the full sample (n = 2500). Cohen (1988) recommends that when Cramer’s V <0.10 this indicates small effects suggesting no practical difference between samples.

^b^ Senior responders included (n = 9) students who self-reported their year in school as fifth or more (undergrad only).

### Procedures

SHIFT employed multiple procedures to assure protection of students involved in our study; these procedures also improve scientific rigor. The study was approved by the Columbia University Medical Center Institutional Review Board and we obtained a federal Certificate of Confidentiality to legally protect our data from subpoena. SHIFT also obtained a University waiver from reporting on individual sexual assaults, as reporting would obviate student privacy and willingness to participate. Students were offered information about referrals to health and mental health resources during the consent process and at the end of the survey, and such information was available from SHIFT via other communication channels. Finally, in reporting data we suppressed data from tables where there were less than 3 subjects in any cell to avoid the possibility of deductive identification of an individual student [44].

SHIFT used principles of Community Based Participatory Research regarding ongoing dialogue with University stakeholders on study development and implementation to maximize the quality of data and impact of research findings [45]. This included weekly meetings between SHIFT investigators and an Undergraduate Advisory Board, consisting of 13–18 students, reflecting the undergraduate student body’s diversity in terms of gender, race/ethnicity, sexual orientation, year in school, and activities (e.g., fraternity/sorority membership). It also included regular meetings with an Institutional Advisory Board comprised of senior administrators, including CU’s Office of General Counsel, facilities, sexual violence response, student conduct, officials involved in gender-based misconduct concerns, athletics, a chaplain, mental health and counseling, residential life, student health, and student life.

Following both the Undergraduate Advisory Board’s recommendations and Dillman’s Tailored Design Method for maximizing survey response rates [43], multiple methods were used to advertise and recruit students. These included: a) email messages, both to generate interest and remind students who had been selected to participate, crafted to resonate with diverse student motives for participation (e.g., interest in sexual assault, compensation, community spirit, and achieving higher response rates than surveys at peer institutions), b) posting flyers, c) holding “study breaks,” in which students were given snacks and drinks, and d) tabling in public areas on campus.

Participants used a unique link to access the survey either at our on-campus research office where computers and snacks were provided (16% of participants) or at a location of their choosing (84% of participants) from March-May, 2016. Before beginning the survey, participants were asked to provide informed consent on an electronic form describing the study, confidentiality, compensation for time and effort, data handling procedures, and the right to refuse to answer any question. Students who completed the survey received $40 in compensation, given in cash to those who completed the survey in our on-campus research office or as an electronic gift card if completed elsewhere. Students were also entered into a lottery to win additional $200 electronic gift cards. This compensation was established based on feedback from student and institutional advisors and reviewed by our Institutional Review Board. It was judged to be sufficient to promote participation, and help ensure that we captured a representative sample, including students who might otherwise have to choose between paid opportunities and participating in our survey, but not great enough to feel coercive for low resource students. This amount of compensation is in line with other similar studies [46]. On average, the survey took 35–40 minutes to complete.

### Measures

The SHIFT survey included behaviorally-specific measures of different types of sexual assault, perpetrated by different methods, as well as measures of key sociodemographic, social and sexual relationship factors, and risk environment characteristics. The majority of instruments had been validated previously with college- age students. The survey was administered in English using Qualtrics (www.qualtrics.com), providing a secure platform for online data collection.

#### Sexual assault

**Fig 1:**
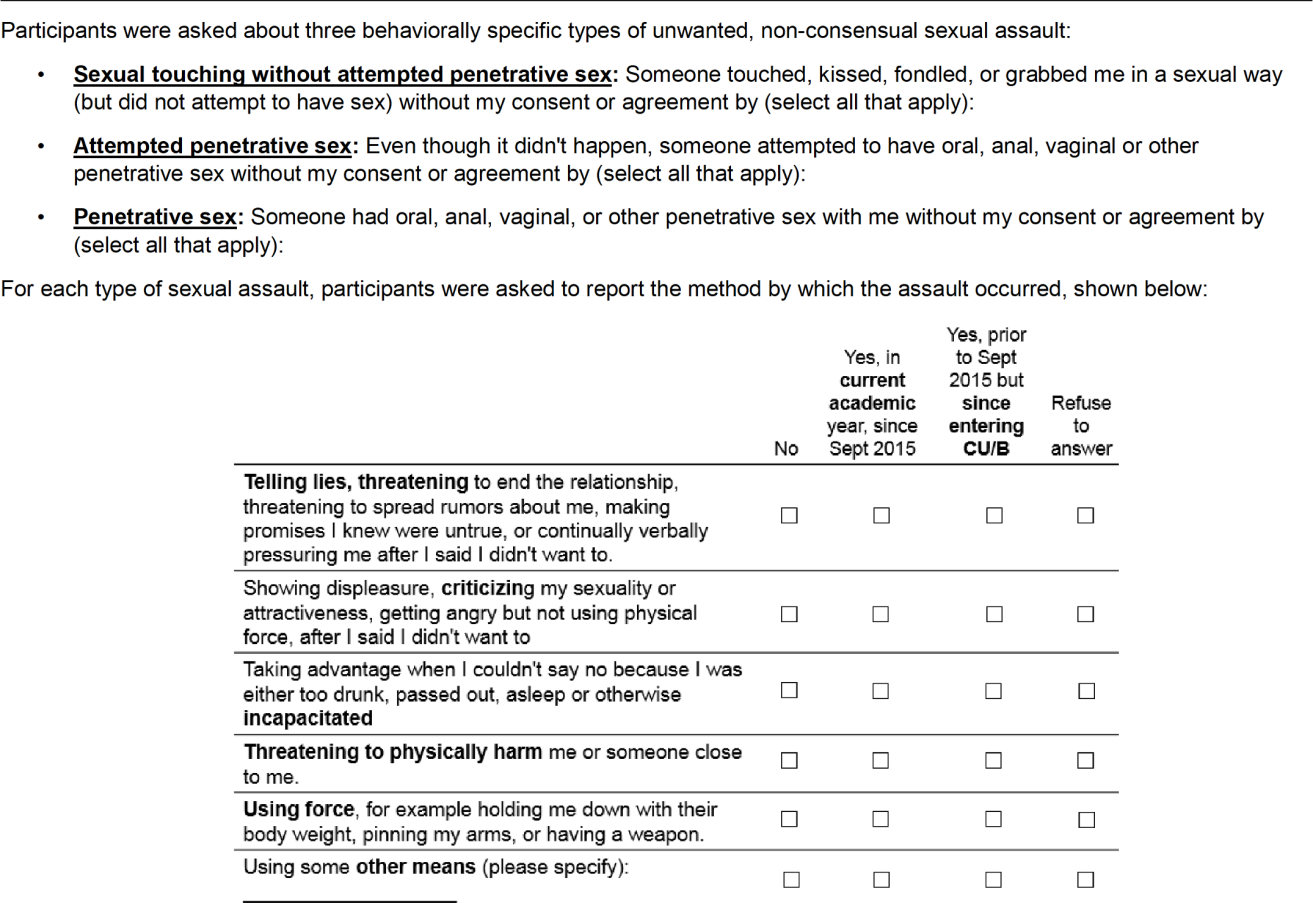
SHIFT survey question on experience of sexual assault.

Sexual assault was assessed with a slightly modified version of the revised Sexual Experiences Survey [16], the most widely used measure of sexual assault victimization with very good psychometric properties including internal consistency and validity previously published [17,47]. The Sexual Experiences Survey employs behaviorally specific questions to improve accuracy [18]. The scale includes questions on type of assault, including sexualized touching without penetration (touching, kissing, fondling, grabbing in a sexual way), attempted but not completed penetrative assault (oral, vaginal, anal or other type of penetration; herein referred to as attempted penetrative assault) and completed penetrative assault (herein referred to as penetrative assault). We used most of the Sexual Experiences Survey as is. However, with strong urging from our Undergraduate Advisory Board, we made a modification, combining the questions about different types of penetration (oral, vaginal, etc.) rather than asking about each kind separately. In the Sexual Experiences Survey, for each type of assault there are six methods of perpetration. Two of the types reflect verbal coercion: 1) “Telling lies, threatening to end the relationship, threatening to spread rumors about me, making promises I knew were untrue, or continually verbally pressuring me after I said I didn’t want to” (herein referred to as “lying/threats”), and 2) “Showing displeasure, criticizing my sexuality or attractiveness, getting angry but not using physical force, after I said I didn’t want to” (herein referred to as “criticism”). The remaining types included use of physical force, threats of physical harm, or incapacitation (“Taking advantage when I couldn’t say no because I was either too drunk, passed out, asleep or otherwise incapacitated”), and other. For each incident of sexual assault, participants could endorse multiple methods of perpetration. Participants were also asked to report whether these experiences occurred: a) during the current academic year (this was a second modification to the Sexual Experiences Survey) and/or b) since enrollment but prior to the current academic year. For this paper, data for the two time periods were combined, reflecting the entire period since starting CU/BC. See Fig 1 for a replica of the questionnaire.

#### Demographics

Demographics included gender identity (male, female, trans-male/trans-female, gender queer/gender-non-conforming, other) [48], year in school (e.g., freshman, sophomore, junior, senior), age, US born (yes/no), lived in US less than five years (yes/no; proxy for recent international student status), transfer student (yes/no), low socioeconomic status (receipt of Pell grant-yes/no [need-based grants for low-income students, with eligibility dependent on family income]); how often participant has trouble paying for basic necessities (never, rarely, sometimes, often, all of the time), and race/ethnicity (non-Hispanic white, non-Hispanic-Asian, non-Hispanic black, Hispanic/Latin-x, other [other included: American Indian or Alaska Native, Native Hawaiian or Pacific Islander, More than one Race/Ethnicity, Other]). Gender was categorized as follows: female, male and GNC (students who responded to gender identity question as anything other than male or female).

#### Fraternity/Sorority

Fraternity/sorority membership (ever participated) was assessed with one question from a school activities checklist (yes/no). We report on Greek life participation here to engage with the substantial attention this has received as a risk factor.

#### Problematic drinking

Problematic drinking during the last year was assessed with the Alcohol Use Disorders Identification Test (AUDIT) [49], a widely used, well-validated standardized 10-item screening tool developed by the World Health Organization. Psychometrics have been established in numerous studies [50–52]. The AUDIT assesses alcohol consumption, drinking behaviors, and alcohol-related problems. Participants rate each question on a 5-point scale from 0 (never) to 4 (daily or almost daily) for possible scores ranging from 0 to 40. The range of AUDIT scores represents varying levels of risk: 0–7 (low), 8–15 (risky or hazardous), 16–19 (high-risk or harmful), and 20 or greater (high-risk). We also examined one AUDIT item on binge drinking, defined as having 6+ drinks on one occasion at least monthly [49].

#### Sexual orientation

Sexual orientation was assessed with one question with the following response options (students could select all that applied): asexual, pansexual, bisexual, queer, heterosexual and homosexual, as well as other [53,54]. Students were categorized into four mutually exclusive groups for analyses: heterosexual, bisexual, homosexual, and other which included asexual, pansexual, queer, or another identity not listed. Non-heterosexual students who indicated more than one orientation were assigned hierarchically to bisexual, homosexual, then other.

#### Romantic/sexual relationships

Romantic/sexual relationships since enrollment at CU/BC were assessed with one question. Response choices included: none, steady or serious relationship, exclusive or monogamous relationship, hook-up-one time, and ongoing hook-up or friends with benefits. Students defined “hookup” for themselves. Students could check all that applied. This variable was trichotomized: at least one hook-up, only steady or exclusive/monogamous relationships, and no romantic/sexual relationships.

#### Pre-college sexual assault

Students also were asked one yes/no question on whether they had experienced any unwanted sexual contact prior to enrolling at CU/BC.

### Data analysis

To assess the representativeness of the sample, the distribution of demographic variables based on administrative records from CU and BC for the total University undergraduate population were compared to the random sample of students contacted, the survey responders, and the current analytic sample, which consists of students that responded to the questions about sexual assault. Demographics for survey responders are based on self-report from the survey. Cramer’s V effect size was used to assess the magnitude of the differences in demographic distributions between the CU/BC population and respondent sample where smaller values (i.e. Cramer’s V <0.10) indicate strong similarity [55].

Analyses were performed on each type of sexual assault as well as a combined “Any type of sexual assault” variable: yes/no experienced sexualized touching, attempted penetrative assault, and/or penetrative assault since CU/BC. Prevalence of each type of sexual assault was calculated by gender and year in school, with chi-square tests of difference used to compare prevalence between genders across each year in school versus freshman year. The total number of incidents of assault and the mean, median and standard deviation for number of incidents of assault per person reporting at least one assault were summarized. Among individuals who experienced any type of sexual assault, the proportions that experienced a particular method of perpetration (e.g. incapacitation, physical force) were calculated by type of sexual assault. Chi-square tests compared proportions between males and females for each perpetration method. The associations of each key correlate with the odds of experiencing any sexual assault were calculated and tested using logistic regression stratified by male/female gender. In addition, a multinomial regression with hierarchical categories (no assault, sexualized touching only, attempted penetrative assault [not completed], and penetrative assault [completed]) as the outcome was performed to examine if associations differed by type of sexual assault. To adjust for the fact that the sample comes from a finite population (i.e. CU/BC N = 5,765 women; N = 3,851 men), a standard finite population correction was implemented for standard error estimation using SAS Proc Surveylogistic. Given the low sample size of GNC students, they were excluded from some analyses. All analyses were conducted using SAS (v. 9.4).

## Results

### Descriptive statistics

Table 1 presents demographic data on the full University, the randomly selected sample, the respondents and the analytic sample for this paper. Among students who consented to the survey (n = 1,671), 46 stopped the survey before the sexual assault questions and 33 refused to answer them resulting in an analytic sample of n = 1,592 (95% completion among responders). Demographic characteristics (i.e. gender [male, female], age, race/ethnicity, year in school, international status, and economic need [Pell grant status]) of the respondent sample were very similar (Cramer’s V effect size differences all <0.10 [55]) to the full CU/BC population (Table 1) indicating that the responder and final analytic samples were representative of the student body population.

The analytic sample included 58% women, 40% men, and 2% GNC students (4 students refused to identify their gender) and was distributed evenly by year in school with most (92%) between18-23 years of age. Self-reported race/ethnicity was 43% white non-Hispanic, 23% Asian, 15% Hispanic/Latino, and 8% black non-Hispanic; 13% were transfer students, and the majority of the sample was born in the US (76%). Twenty-three percent of participants received Pell grants and 51% of students acknowledged at least sometimes having difficulty paying for basic necessities.

The majority of women (79%) and men (85%) identified as heterosexual. In terms of romantic/sexual relationships since starting CU/BC, 30.0% of women and 21.6% of men reported no relationships, 21.0% of women and 22.6% of men reported only steady/exclusive relationships with no hookups, and 49.0% of women and 55.7% of men reported at least one hook-up. Finally, 25.5% of women, 9.4% of men, and 47.0% of GNC students reported pre-college sexual assault.

### Aim 1: Prevalence of sexual assault victimization at CU/BC

#### Overall rates by gender and school year

Since starting CU/BC, 22.0% (350/1,592) of students reported experiencing at least one incident of any sexual assault across the three types (sexualized touching, attempted penetrative assault, and penetrative assault). Table 2 presents data on types of assault by gender and year in school. Women were over twice as likely as men to report any sexual assault (28.1% vs 12.5%). There was evidence of cumulative risk for experiencing sexual assault among women over four years of college, so that by junior and senior year, respectively, 29.7% and 36.4% of women reported experiencing any sexual assault, compared to 21.0% of freshman women who had only one year of possible exposure (p < .05). However, one-fifth (21.0%) of women who took the survey as freshman had experienced unwanted sexual contact, compared to 36.4% over 3+ years (seniors), suggesting that as others have found, the risk of assault is highest in freshman year.

**Table 2:** Sexual assault since enrolling at CU/BC, by respondent gender and year in school.

	Any type of sexual assault		Sexualized touching		Penetrative assault		Attempted penetrative assault	
	n	%	n	%	n	%	n	%
**Female (N = 928)**	261	28.1	219	23.6	126	13.6	103	11.2
Freshman (N = 224)	47	21.0	42	18.8	17	7.6	12	5.4
Sophomore (N = 231)	59	25.5	49	21.2	26	11.3	21	9.2
Junior (N = 246)	73	29.7*	57	23.0	37	15.0*	35	14.2*
Senior (N = 225)	82	36.4*	71	31.6*	46	20.5*	35	15.7*
**Male (N = 634)**	79	12.5	70	11.0	33	5.2	24	3.8
Freshman (N = 162)	16	9.9	15	9.3	5	3.1	-	-
Sophomore (N = 150)	16	10.7	12	8.0	5	3.3	6	4.0
Junior (N = 161)	22	13.7	19	11.8	13	8.1*	8	5.0*
Senior (N = 160)	25	15.6	24	15.0	10	6.3	9	5.6*
**GNC (N = 26)**	10	38.5	10	38.5	-	-	-	-

Note: Some respondents reported multiple unique incidents corresponding to multiple types of unwanted sexual contact; therefore, total number of respondents who experienced each of the three types of unwanted sexual contact do not sum to total number of respondents who experienced "Any type" of unwanted sexual contact.

* p < .05 for test of proportion difference vs. Freshman within each gender.

Cells with 3 or fewer respondents have been suppressed, noted here with a dash through the cell.

Among men, one in eight indicated that they had been sexually assaulted since starting CU. Similar to women, the risk for sexual assault among men accumulated over the four years of college, with 15.6% of seniors vs 9.9% of freshman reporting a sexual assault since entering CU, although this difference was not statistically significant.

Although the numbers were small, GNC students reported the highest prevalence of sexual assault since starting CU/BC (38.5%; 10/26). Numbers were too small (n<3) to present stratified by year in school (see Table 2).

#### Types of sexual assault by gender (Table 2)

The most prevalent form of sexual assault was sexualized touching; rates for women (23.6%) and GNC students (38.5%) were significantly higher than rates for men (11.0%; p < .05). Prevalence of attempted penetrative assault and penetrative assault were about half that of sexualized touching. Compared to men, women were three times as likely to report attempted penetrative assault (11.1% vs 3.8%) and over twice as likely to experience penetrative assault (13.6% vs 5.2%). Among GNC students, the majority reporting sexualized touching, with rates of the other two types too small to report.

#### Experiencing multiple sexual assaults (Fig 2; S1 Table)

Students could report multiple types of sexual assault incidents (i.e. sexualized touching, attempted penetrative, and penetrative assault) as well as multiple incidents experienced of each type. Overall, students reported a total of 1,007 incidents of sexual assault experienced since starting CU/BC. For the 350 students who indicated any sexual assault, the median number of incidents experienced was 3.

**Fig 2:**
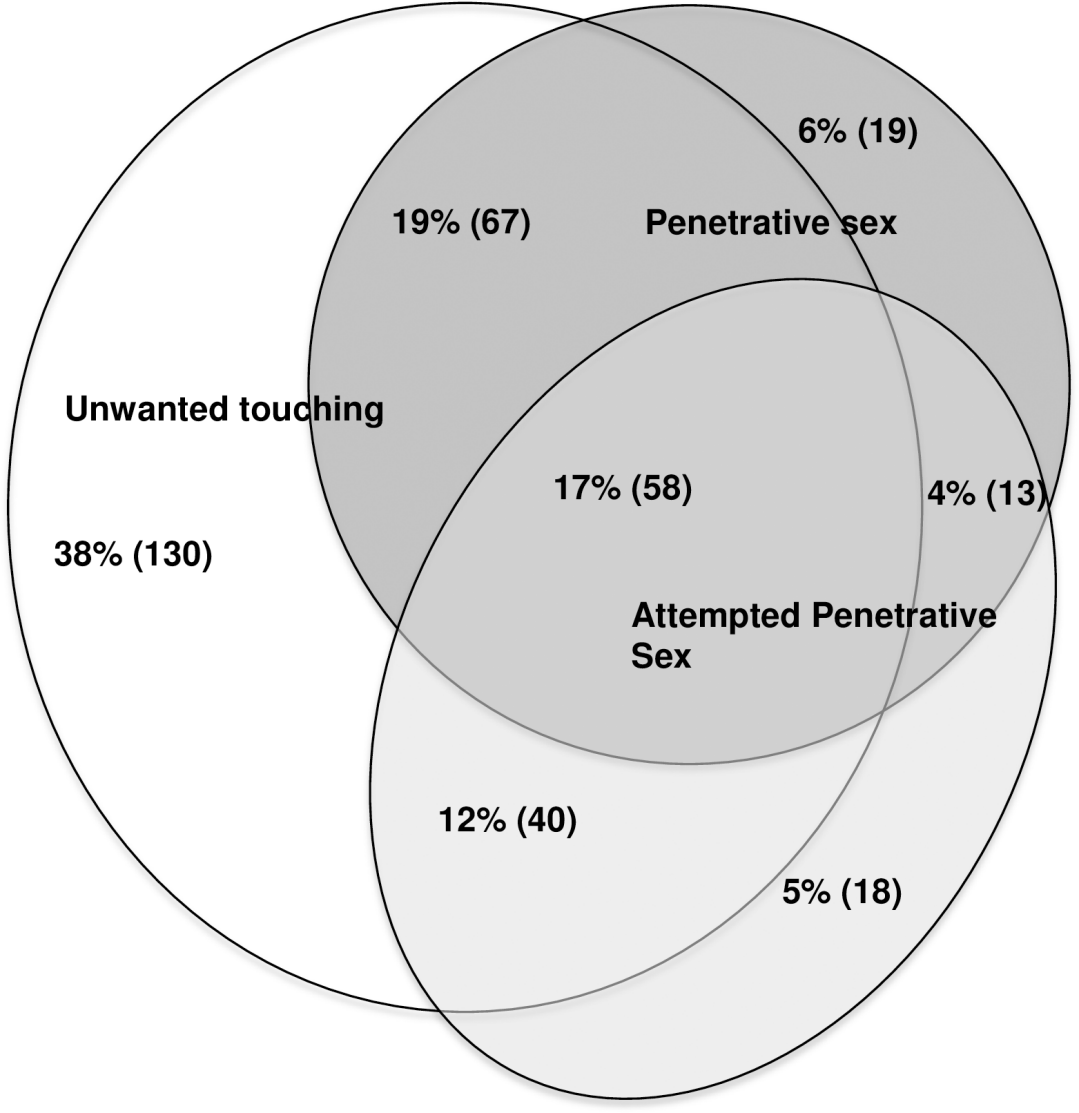
Overlap of different types of sexual assaults experienced by n = 350 students (all genders).

Among the 350 students reporting any sexual assault, Fig 2 presents different combinations of sexual assault experienced by students since CU/BC. Most prevalent, 38.0% reported experiencing only sexualized touching; 19.0% reported both sexualized touching and penetrative assault incidents; 17.0% experienced all three types of assault; and 12.0% sexualized touching and attempted penetrative assault.

### Aim 2: Methods of perpetration (lying/threats, criticism, incapacitation, physical force, threats of harm, and other) by gender (Table 3)

Across types of assault, incapacitation was the method of perpetration reported most frequently (> 50%) in both men and women. For both women and men, approximately two-thirds of all penetrative assaults and about half of sexualized touching and attempted penetrative assaults involved incapacitation.

**Table 3:** Methods of perpetration used in sexual assaults since enrolling at CU/BC, by type and gender.

	Any type of sexual assault		Sexualized touching		Penetrative assault		Attempted penetrative assault	
	n	%^a^	n	% ^a^	n	% ^a^	n	% ^a^
**Female**	261		219		126		103	
Lied or threatened	80	30.7	57	26.0	34	27.0	41	39.8
Criticized	99	37.9	77	35.2	43	34.1	44	42.7
Took advantage while incapacitated	148	57.1	118	54.1	82	65.1	49	47.6
Threatened physical harm	9	3.5	7	3.2	4	3.2	-	-
Used physical force	90	34.6*	70	32.1*	42	33.3	28	27.2
Did something else	32	12.5	22	10.1	10	8.0	4	3.9
**Male**	79		70		33		24	
Lied or threatened	22	27.8	17	24.3	11	33.3	7	29.2
Criticized	27	34.2	21	30.0	7	21.2	8	33.3
Took advantage while incapacitated	43	54.4	36	51.4	22	66.7	13	54.2
Threatened physical harm	-	-	-	-	-	-	-	-
Used physical force	10	12.7*	7	10.0*	-	-	4	16.7
Did something else	8	10.1	8	11.4	-	-	-	-

* p < .05 for test of proportion difference between male vs female for specific method of coercion by type.

^a^ % can add up to more than 100% within type due to multiple coercion methods reported.

Cells with 3 or fewer respondents have been suppressed, noted here with a dash through the cell.

Physical force was reported significantly more frequently by women than men (34.6% vs 12.7%) for any sexual assault. More specifically, compared to men, women were three times more likely to experience sexualized touching via physical force (32.1% vs. 10.0%), and six times more likely to experience penetrative assaults via physical force (33.3% vs 6.1%).

Lastly, a sizeable number of respondents reported verbal coercion (ranging from 21.0% to over 40.0% depending on type of assault). Criticism was cited by women at rates similar to physical force for both sexualized touching and penetrative assaults. Among men, both verbal coercion methods were cited most frequently after incapacitation for all three types of assault.

For GNC students, we examined rates of each perpetration method for only the composite variable any sexual assault (due to small numbers in any specific type of assault). Among those who experienced an assault, incapacitation was the most frequently mentioned method (50.0%), followed by criticism (40.0%).

### Aim 3: Identify factors associated with sexual assault experiences

We examined the association between sexual assault (both any sexual assault [Table 4] and each type of sexual assault [Table 5]) and key demographic, sexual history and social activity factors. Results are stratified by gender (women/men).

**Table 4:** Associations between demographic and behavioral characteristics and experience of any sexual assault since enrolling at CU/BC, by gender.

			Female			Male		
			Sample distribution (N = 928)		Odds of experiencing any sexual assault (n = 261)	Sample distribution (N = 634)		Odds of experiencing any sexual assault (n = 79)
			n	%	OR (95% CI)	n	%	OR (95% CI)
***Demographic characteristics***								
	**Race/ethnicity**							
		Non-Hispanic White	388	42.5%	REF	280	45.2%	REF
		Asian	213	23.4%	**0.58 (0.40–0.84)**	139	22.5%	**0.46 (0.24–0.87)**
		Black	85	9.3%	1.29 (0.82–2.04	43	6.9%	0.55 (0.20–1.48)
		Hispanic	133	14.6%	1.02 (0.69–1.52)	102	16.5%	0.78 (0.42–1.44)
		Other	93	10.2%	0.97 (0.61–1.54)	55	8.9%	0.54 (0.22–1.31)
	**Difficulty paying for basic necessities**^**3**^							
		Never	459	49.9%	REF	352	56.0%	REF
		Rarely or sometimes	389	42.3%	**1.48 (1.12–1.96)**	242	38.5%	1.19 (0.75–1.87)
		Often or all of the time	71	7.7%	**1.86 (1.14–3.01)**	35	5.6%	1.61 (0.68–3.82)
	**Transfer student**							
		Yes	123	13.3%	**0.58 (0.38–0.89)**	104	16.4%	0.80 (0.43–1.49)
		No	804	86.7%	REF	529	83.6%	REF
	**Sexual Identity**							
		Heterosexual	726	78.8%	REF	529	85.0%	REF
		Homosexual	20	2.2%	0.71 (0.26–1.95)	54	8.7%	**3.02 (1.63–5.85)**
		Bisexual	102	11.1%	**1.61 (1.08–2.40)**	26	4.2%	2.05 (0.81–5.21)
		Other	73	7.9%	**1.76 (1.11–2.78)**	13	2.1%	2.59 (0.77–8.69)
	**Lived in US less than 5 years**							
		Yes	89	9.6%	0.77 (0.48–1.23)	75	11.9%	0.47 (0.20–1.11)
		No	836	90.4%	REF	554	88.1%	REF
***Behavioral correlates***								
	**Relationship status, since CU/BC**							
		Steady/exclusive, no hook-ups	189	21.0%	REF	138	22.6%	REF
		None	270	30.0%	0.64 (0.39–1.06)	132	21.6%	1.62 (0.43–6.14)
		Hook-ups	441	49.0%	**4.28 (2.87–6.37)**	340	55.7%	**10.94 (3.72–32.22)**
	**Fraternity/sorority participation**							
		Yes	164	18.2%	**1.46 (1.05–2.03)**	149	24.1%	**1.82 (1.14–2.90)**
		No	737	81.8%	REF	470	75.9%	REF
	**AUDIT item on binge drinking (6+ drinks on one occasion) at least monthly**							
		Yes	165	18.0%	**2.72 (1.98–3.75)**	247	40.1%	**1.59 (1.02–2.46)**
		No	752	82.0%	REF	369	59.9%	REF
	**AUDIT total score on risky or hazardous drinking**^**a**^							
		Yes	177	19.4%	**4.04 (2.95–5.54)**	238	38.8%	**2.36 (1.51–3.69)**
		No	736	80.6%	REF	376	61.2%	REF
**Experienced unwanted sexual contact prior to CU/BC**								
		Yes	233	25.5%	**2.52 (1.88–3.37)**	59	9.4%	**2.20 (1.19–4.05)**
		No	679	74.5%	REF	570	90.6%	REF

^a^As measured by a score of 8 or more on the Alcohol Use Disorders Identification Test (AUDIT).

**Table 5:** Associations between demographic and behavioral characteristics and each type of sexual assault since enrolling at CU/BC, by gender.

			Female (N = 928)			Male (N = 634)		
			*Penetrative vs*. *no assault*	*Attempted penetrative vs*. *no assault*	*Touching only vs*. *no assault*	*Penetrative vs*. *no assault*	*Attempted penetrative vs*. *no assault*	*Touching only vs*. *no assault*
			OR (95% CI)	OR (95% CI)	OR (95% CI)	OR (95% CI)	OR (95% CI)	OR (95% CI)
***Demographic characteristics ***								
	**Race/ethnicity**							
		Non-Hispanic White	REF	REF	REF	REF	REF	REF
		Asian	**0.35 (0.19–0.62)**	0.56 (0.25–1.26)	1.00 (0.59–1.69)	0.33 (0.10–1.03)	1.11 (0.29–4.23)	0.42 (0.17–1.05)
		Black	0.99 (0.53–1.87)	0.99 (0.35–2.78)	**1.99 (1.05–3.74)**	0.71 (0.18–2.87)	NE	0.55 (0.14–2.18)
		Hispanic	1.08 (0.65–1.78)	1.32 (0.62–2.82)	0.75 (0.37–1.54)	1.25 (0.55–2.81)	1.06 (0.23–4.94)	0.36 (0.12–1.13)
		Other race / >1 race /ethnicity	1.10 (0.63–1.95)	1.03 (0.40–2.65)	0.71 (0.31–1.64)	0.56 (0.14–2.22)	0.94 (0.13–7.05)	0.43 (0.11–1.69)
	**Difficulty paying for basic necessities**^**3**^							
		Never	REF	REF	REF	REF	REF	REF
		Rarely or sometimes	**1.47 (1.01–2.14)**	1.26 (0.71–2.22)	**1.65 (1.07–2.55)**	1.59 (0.80–3.17)	0.85 (0.27–2.66)	0.99 (0.45–2.17)
		Often or all of the time	**2.24 (1.23–4.09)**	1.06 (0.34–3.34)	1.78 (0.83–3.81)	**3.07 (1.04–9.07)**	NE	1.07 (0.42–2.73)
	**Transfer student**							
		Yes	0.60 (0.34–1.08)	1.03 (0.48–2.21)	**0.34 (0.15–0.80)**	0.89 (0.36–2.17)	1.10 (0.27–4.57)	0.64 (0.24–1.70)
		No	REF	REF	REF	REF	REF	REF
	**Sexual Identity**							
		Heterosexual	REF	REF	REF	REF	REF	REF
		Homosexual	0.74 (0.19–2.94)	1.05 (0.16–6.95)	0.49 (0.08–3.22)	**4.74 (2.10–10.71)**	1.18 (0.17–8.11)	2.37 (0.93–6.05)
		Bisexual	1.56 (0.91–2.66)	2.06 (0.97–4.38)	1.45 (0.89–2.69)	**3.39 (1.03–11.16)**	NE	1.81 (0.44–7.26)
		Other	**2.11 (1.20–3.73)**	1.86 (0.75–4.64)	1.23 (0.57–2.65)	**4.74 (1.10–20.48)**	NE	1.90 (0.27–13.15)
	**Lived in US less than 5 years**							
		Yes	0.52 (0.25–1.08)	0.61 (0.20–1.84)	1.24 (0.66–2.31)	NE	1.53 (0.37–6.36)	0.66 (0.22–2.02)
		No	REF	REF	REF	REF	REF	REF
***Behavioral correlates***								
	**Relationship status, since CU/BC**							
		Steady/exclusive, no hook-ups	REF	REF	REF	REF	REF	REF
		None	**0.05 (0.01–0.31)**	0.66 (0.21–2.10)	1.38 (0.69–2.75)	NE	NE	3.88 (0.51–29.8)
		Hook-ups	**5.03 (2.91–8.68)**	**4.43 (1.83–10.8)**	**3.26 (1.74–6.09)**	NE	2.14 (0.51–8.94)	**13.3 (2.09–85.1)**
	**Fraternity/sorority participation**							
		Yes	1.39 (0.90–2.15)	1.34 (0.66–2.70)	**1.63 (1.00–2.67)**	1.29 (0.62–2.67)	1.96 (0.62–6.17)	**2.40 (1.25–4.63)**
		No	REF	REF	REF	REF	REF	REF
	**AUDIT item on binge drinking (6+ drinks on one occasion) at least monthly**							
		Yes	**3.12 (2.09–4.65)**	**2.28 (1.20–4.33)**	**2.42 (1.50–3.91)**	**2.15 (1.12–4.15)**	2.78 (0.89–8.70)	0.98 (0.51–1.89)
		No	REF	REF	REF	REF	REF	REF
	**AUDIT total score on risky or hazardous drinking**^**a**^							
		Yes	**6.04 (4.10–8.90)**	**3.38 (1.84–6.19)**	**2.33 (1.42–3.81)**	**4.07 (2.01–8.21)**	3.09 (0.99–9.71)	1.30 (0.68–2.51)
		No	REF	REF	REF	REF	REF	REF
**Experienced unwanted sexual contact prior to CU/BC**								
		Yes	**3.01 (2.07–4.37)**	**3.74 (2.10–6.66)**	1.55 (0.98–2.46)	**2.44 (1.03–5.76)**	1.10 (0.16–7.41)	**2.35 (1.00–5.54)**
		No	REF	REF	REF	REF	REF	REF

NE = Not estimable due to small cell sizes.

^a^As measured by a score of 8 or more on the Alcohol Use Disorders Identification Test (AUDIT).

#### Race/Ethnicity

For both women and men, the prevalence of any sexual assault was similar for all race/ethnicity groups compared to non-Hispanic White students with one exception. Asian students (women and men) were less likely to experience any sexual assault than non-Hispanic White students. For women only, differences emerged by type of assault. Asian women compared to non-Hispanic White women were less likely to experience penetrative assault (OR = 0.35, CI: 0.19–0.62), but not attempted penetrative assault (OR = 0.56, CI: 0.25–1.26), nor sexualized touching only (OR = 1.00, CI: 0.59–1.69). Black women were found to have increased odds of touching only incidents compared to non-Hispanic White women (OR = 1.99, CI: 1.05–3.74). There were no other significant racial or ethnic differences.

#### Economic precarity

Women who often or always had difficulty paying for basic necessities had increased odds of any sexual assault; for men the trend was similar but it did not reach statistical significance. Considering penetrative assault specifically, both men and women who often or always had difficulty paying for basic necessities had increased risk (women OR = 2.24, CI: 1.23–4.09; men OR = 3.07, CI: 1.04–9.07) compared to those who never had difficulty.

#### Transfer student

Women transfer students were less likely to experience any sexual assault than non-transfer students. Closer inspection of type of assault revealed that this protective effect was seen for sexualized touching only (OR = 0.34, CI: 0.15–0.80), but not for penetrative (OR = 0.60, CI: 0.34–1.08), nor attempted penetrative (OR = 1.03, CI: 0.48–2.21) assault. There were no significant differences between men who were transfer students and those who were not.

#### Sexual orientation

For women, those who identified as bisexual and those who identified as some other sexual identity besides heterosexual, homosexual, or bisexual (includes people endorsing exclusively one or a combination of: Asexual, Pansexual, Queer, or a sexual orientation not listed), were more likely to experience any sexual assault than heterosexual students. For penetrative assault specifically, this increased risk was only present for individuals with some other sexual identity (OR = 2.11, CI: 1.20–3.73). For men, those who identified as homosexual were more likely to experience any sexual assault than heterosexual male students. For penetrative assault specifically, those who identified as homosexual, bisexual, or some other sexual identity all had substantially increased risk compared to those with a heterosexual identity (OR = 4.74, CI: 2.10–10.71; OR = 3.39, CI: 1.03–11.16; OR = 4.74, CI:1.10–20.48, respectively).

Information about the gender of the perpetrator for different gender and sexual orientation groups was available for a subset of incidents (336/997). Among these events, 98.4% (3/184) of the heterosexual women indicated the perpetrator was a man, while 97.1% (33/34) of the bisexual women, 75% (3/4) of the homosexual women, and 88.9% (24/27) of the other sexual identity women indicated it was a man. For men who were assaulted, 84.9% (45/53) of the heterosexual men reported the perpetrator was a woman, while 0 of the homosexual men said the perpetrator was a woman. Numbers for bisexual men and other sexual identity men were too small to report separately, but combined showed that 5/8 (63.0%) of bisexual and other sexual identity men said the perpetrator was a woman. Of the GNC students reporting on a most-significant event, 77.8% (7/9) reported that they were assaulted by a male perpetrator (the numbers are too small to further examine by sexual orientation).

#### Lived in US less than 5 years

There was no association found between living in the US for less than 5 years and any sexual assault, nor any specific type of sexual assault.

#### Relationship status

Among both women and men, students who had at least one hook-up were more likely to have experienced any sexual assault than students who were in only steady/exclusive relationships since starting college. Among women who had engaged in at least one hook-up, this increased risk held for each type of sexual assault (penetrative: OR = 5.03, CI = 2.91–8.68, attempted penetrative: OR = 4.43, CI = 1.83–10.8, sexualized touching only: OR = 3.26, CI = 1.74–6.09), while among men the increased risk was found for sexualized touching only (OR = 13.33, CI = 2.09–85.08), but could not be estimated (due to small numbers) for completed penetrative assault. Women who did not have any romantic or sexual relationship since CU/BC were found to be less likely to experience penetrative assault than women who had a steady/exclusive relationships only (OR = 0.05, CI: 0.01–0.31).

#### Fraternity/Sorority membership

Although a relative minority of students participated in fraternities (24.1%) or sororities (18.2%), for both men and women, those who participated were more likely to experience any sexual assault than those who did not. Examination of type of assault revealed that the effect is driven primarily by sexualized touching only which is significant in both women (OR = 1.63, CI: 1.00–2.67) and men (OR = 2.40, CI: 1.25–4.63) and not significantly increased for penetrative nor attempted penetrative assault.

#### Risky or hazardous drinking

For both men and women, individuals who met criteria on the AUDIT for risky or hazardous drinking were more likely to experience any sexual assault than those who did not. When examining each type of assault separately, for men this increased risk was only significant for penetrative assault (OR = 4.07, CI: 2.01–8.21). For women, the increased risk of assault held for each type of assault—penetrative (OR = 6.04, CI: 4.10–8.90), attempted (OR = 3.38, CI: 1.84–6.19) and touching (OR = 2.33, CI: 1.42–3.81). We also looked at one AUDIT item specifically on binge drinking (6 or more drinks on a single occasion). Individuals who reported binge drinking at least monthly were more likely to experience any sexual assault than those who did not. When examining each type of assault separately, for men this increased risk was only significant for penetrative assault (OR = 2.15, CI: 1.12–4.15). For women, this increased risk was significant for penetrative assault (OR = 3.12, CI: 2.09–4.65), attempted assault (OR = 2.28, CI: 1.20–4.33), and touching (OR = 2.42, CI:1.50–3.91).

#### Pre-college assault (Table 5)

Among both women and men, those who experienced pre-college assault were more likely to experience any sexual assault while at CU/BC. The increased risk held for penetrative assault in both women (OR = 3.01, CI: 2.07–4.37) and men (OR = 2.44, CI: 1.03–5.76). In women, the increased risk also held for attempted penetrative, but not touching only, whereas in men, the increased risk held for touching only, but not attempted penetrative sex.

## Discussion

The SHIFT survey, with a population-representative sample, good response rate and behaviorally-specific questions, found that 22.0% of students reported a sexual assault since starting college, which confirms previous studies of 1 in 4 or 1 in 5 prevalence estimates with national samples and a range of types of schools [23,24]. However, a key finding is that focusing only on the “1 in 4/ 1 in 5” rate of any sexual assault obscures much of the nuance concerning types of sexual assault as well as the differential group risk, as prevalence rates were unevenly distributed across gender and several other social and demographic factors.

Similar to other studies [4,24], women had much higher rates of experiencing any type of sexual assault compared to men (28.0% vs 12.0%). Moreover, our data suggest a cumulative risk for sexual assault experiences over four years of college with over one in three women experiencing an assault by senior year. However, our data also suggest that freshman year, particularly for women, is when the greatest percentage experience an assault. This supports other work on freshman year as a particularly critical time for prevention efforts, otherwise known as the “red zone” effect for women [32].

Importantly, our study confirms that GNC students are at heightened risk for sexual assault [23]. They had the highest proportion of sexual assaults, with 38.0% reporting at least one incident, the majority of which involved unwanted/non-consensual sexualized touching. These data should be interpreted very cautiously given the small number of GNC students. However, increasingly studies suggest that transgender and other GNC students have sexual health needs that may not be targeted by traditional programming [57]; thus, a better understanding of pathways to vulnerability among these students is of high importance.

Similarly, students who identified as a sexual orientation other than heterosexual were at increased risk for experiencing any sexual assault, with bisexual women or women who identified as “other” and men who identified as any non-heterosexual category at increased risk. Similar to GNC students, understanding the specific social and sexual health needs of LGB students, particularly as it relates to reducing sexual assault risk is critical to prevention efforts [58]. Factors such as stigma and discrimination, lack of communication, substance use, as well as a potential lack of tailored prevention programs may play a role. To our knowledge, there are no evidence-based college sexual assault prevention programs targeting LGB and GNC students. Our data suggest that the LGB and GNC experiences are not uniform; more research should be done within each of these groups to understand the mechanisms behind their potentially unique risk factors.

Our data also suggest that the 20–25% rate of any sexual assault obscures variation in assault experiences. Sexualized touching accounted for the highest percentage of acts across gender groups, with over one-third of participants reporting only sexualized touching incidents. Rates of attempted and completed penetrative sexual assault were about half the rate of sexualized touching. This finding does not minimize the importance of addressing unacceptably high rates of attempted penetrative and penetrative assault (14%-15%), but it does suggest the importance of specificity in prevention efforts. For GNC students, for example, the risk of assault was primarily for sexualized touching with very few reporting attempted penetrative assault or penetrative assault during their time at CU/BC. These elevated rates of unwanted sexual touching may be a combination of GNC students’ focus on their gendered sexual boundaries–and thus potentially greater awareness of when advances are unwanted–at a developmental moment when they are building non-traditional gender identities, as well as these students’ social vulnerability. Further investigation is warranted.

Moreover, there was variation in methods of perpetration reported by survivors of sexual assault. Incapacitation was the most common method reported across all gender groups for each type of assault, and female and male students who reported risky or hazardous drinking were at increased risk for experiencing any sexual assault, particularly penetrative assault. Across campuses in the US, hazardous drinking is a national problem with substantive negative health outcomes, risk for sexual assault being one of them [2,39,59]. Our data underline the potential of programs and policies to reduce substance use and limit its harms as one element of comprehensive sexual assault prevention; we found few evidence-based interventions that address both binge drinking and sexual assault prevention. Of course, any work addressing substance use as a driver of vulnerability must do so in a way that does not replicate victim-blaming.

However, similar to other studies with broad foci, incapacitation was not the only method of perpetration reported. For women, physical force, particularly for penetrative sex, was the second most frequently endorsed method. Verbal coercion, including criticism, lying and threats to end the relationship or spread rumors, was also employed at rates similar to physical force for women, and was the second most frequently endorsed category for men and GNC students. Prevention programs, such as the bystander interventions which are the focus of efforts on many campuses [60], often focus on incapacitation or physical force. These interventions tend to highlight situations where survivors (typically women) are vulnerable because they are under the influence of substances. In SHIFT, verbal coercion is also shown to be a powerful driver of assault; however, it typically does not receive as much attention as rape, which is legally defined as penetration due to physical force or incapacitation. If a survivor is verbally coerced into providing affirmative consent, the incident could be considered within consent guidelines of “yes means yes” but it may have been unwanted by the survivor [61,62]. Assertiveness interventions and those that focus on verbal consent practices may be useful for addressing this form of assault.

We also found high rates of re-victimization. As others have found, pre-college sexual assault was a key predictor for experiencing assault at CU/BC [33,36]. However, we also found high rates of repeat victimization since starting at CU/BC with a median of 3 incidents per person reporting any sexual assault since starting CU/BC, and the highest risk of repeat victimization in women and GNC students. These data underline the importance of prevention efforts that include care for survivors to reduce the enhanced vulnerability that has been shown in other populations of assault survivors [36]. Future studies should also seek to disaggregate the relationship between type of victimization (sexualized touching, attempted penetrative assault, penetrative assault) and repeat victimization.

This study also identified a number of variables associated with sexual assault, some similar to previous studies and others different. As noted, gender was a key correlate. While prevention efforts should respond to the population-level burden by focusing on the needs of women and GNC students, it is important to note that men were also at risk of sexual assault. In our study, nearly 1 in 8 men reported a sexual assault experience, a rate also found in the Online College Social Life survey [56], but higher than other studies [63,64]. Few programs target men, and issues around masculinity and gender roles may make it difficult for men to consider or report what has happened to them as sexual assault. Importantly, this study found that men who were members of fraternities were at higher risk for experiencing assault (specifically unwanted/nonconsensual sexualized touching) than those who were not members. This is consistent with previous findings, including the Online College Social Life survey [56], but is of particular note because research has identified men in fraternities as more likely to be perpetrators [64], but few, if any, studies have looked at fraternity members’ vulnerability to sexual assault. Our data suggest a need for further examination of the cultural and organizational dimensions of Greek life that produce this heightened risk of being assaulted for both men and women. However, it is important to note that we did not examine a range of other social and extracurricular groups which may have produced risk as well and thus a more full examination of student undergraduate life is needed.

One other key factor associated with assault was participation in “hook ups”. Both male and female students who reported hooking up were more likely to report experiencing sexual assault, compared to students who only had exclusive or monogamous relationships and those who had no sexual relationships. The role of hooking up on college campuses has received much attention in the popular press and in a number of books [65,66], but little has been written about its connection to sexual assault, although several recent studies are in line with ours about its role as a risk factor for experiencing sexual assault on college campuses [40,41]. Multiple mechanisms may be at work: students who participate in hookups may be having sex with more people, and thus face greater risk of assault due to greater exposure to sex with a potential perpetrator, but students who participate in hookups may also face increased vulnerability because many hookups involve “drunk” sex, or because hookups by definition involve sexual interactions between people who are not in a long-term intimate relationship, and thus whose bodies and social cues maybe unfamiliar to each other. Alternatively some aspects of hook-ups may be more or less risky than others and therefore continued study of different dimensions of these more casual relationships that can refer to a wide-range of behaviors is necessary.

Several demographic characteristics were not for the most part associated with sexual assault. We did not find racial or ethnic differences in sexual assault risk with primarily one exception, Asian male and female students were at less risk overall compared to white students. We also did not find transfer students to be at greater risk; female transfer students were actually at lower risk, potentially due to less exposure time, particularly during freshman year. International student status as indicated by having been in the US<5 years was also not associated with increased risk. However, this study highlights the role of economic factors that have received limited attention in the literature. Little is known about how economic insecurity may drive vulnerability, but issues of power, privilege, and control of alcohol and space all require further examination.

There are several limitations to this study. Participants came from only two private schools that are interconnected in one city, and thus findings may not generalize to the rest of the US. There is a continued need for more national studies with different types of colleges and universities in urban and rural environments with more varied economic backgrounds in order to fully understand institutional and contextual differences. Although we had a response rate that was higher than many prior studies and our rates of sexual assault are consistent with prior studies [4], we cannot assess the extent to which selection bias may have occurred and therefore, our rates could be an underrepresentation or overrepresentation depending on who chose to participate. Although this concern is somewhat mitigated by findings that basic demographic data between respondents and the total population of students at two colleges suggest no significant differences, there may be some bias in factors we did not consider. Our present analysis has focused only on bivariate associations between risk factors and assault. While this analysis provides a valuable description of which groups are at elevated risk or not, future work will consider how combinations of risk factors at different levels may interact to increase risk. Critically, the analysis presented here reflects a focus on those who experience being assaulted, but in other work we look at the characteristics of perpetrators, both from those who reported perpetrating and from a subset of incidents that survey respondents described in depth, which provided more information about the perpetrator. A greater understanding of the characteristics and contexts of perpetration is without question vital for effective prevention. Finally, our data are cross sectional. Longitudinal studies with a comprehensive range of predictors are critical for identifying pathways of causality and targets for interventions.

Despite these limitations, this study confirms the unacceptably high rates of sexual assault and suggests diversity in experiences and methods of perpetration. A key conclusion is that a”one size fits all” approach that characterizes the extant literature on evidence-based prevention programs [67] may need to be altered to more effectively prevent sexual assault in college. Clearly different groups had differential risk for assault and may require much more targeted prevention efforts. Bystander interventions have shown promise in addressing risk in social situations, including fraternity parties and other settings with high alcohol use [68,69]. However, bystander interventions may not be sufficient for incidents occurring in non-party contexts where verbal coercion methods or physical force may be used without others around.

Creating effective and sustainable changes to campus culture requires engaging with a broad range of institutional stakeholders. SHIFT investigators are in the process of sharing selected findings with both student and institutional advisory boards, and an intensive collaborative process allows us to explore the implications of our results for a broad range of policies and programs, including both elements commonly considered as sexual assault prevention (consent education, bystander trainings), more general topics related to sexual orientation and verbal discussions of sex, and aspects of the institutional context across diverse domains including alcohol policy, mental health services, residential life policies, orientation planning, and the allocation of space across campus.

Overall, our findings argue for the potential of a systems-based [70] public health approach–one that recognizes the multiple interrelated factors that produce adverse outcomes, and perhaps particularly emphasizes gender and economic disparities and resulting power dynamics, widespread use of alcohol, attitudes about sexuality, and conversations about sex–to make inroads on an issue that stubbornly persists.

## Supporting information

S1 Table Number of incidents of sexual assault since enrolling at CU/BC, among individuals with at least one incident. (DOCX)
