# Correction: Sexual assault incidents among college undergraduates: Prevalence and factors associated with risk

**DOI:** 10.1371/journal.pone.0192129

**Published:** 2018-01-25

**Authors:** The PLOS ONE Staff

There is an error in the Funding section. The correct funding information is as follows: This research was funded by Columbia University through a donation from the Lavine Family. The funder (Lavine Family) had no role in study design, data collection and analysis, decision to publish, or preparation of the manuscript. The publisher apologizes for the error.

## References

[1] Mellins CA, Walsh K, Sarvet AL, Wall M, Gilbert L, Santelli JS, et al (2017) Sexual assault incidents among college undergraduates: Prevalence and factors associated with risk. PLoS ONE 12(11): e0186471 https://doi.org/10.1371/journal.pone.0186471 29117226 10.1371/journal.pone.0186471 PMC5695602

