# Supplementary material for: Sexual assault incidents among college undergraduates: Prevalence and factors associated with risk
Source: PLoS One. 2017 Nov 8;12(11):e0186471. doi: 10.1371/journal.pone.0186471 (PMC5695602; doi:10.1371/journal.pone.0186471)
Supplement: S1 Table — (DOCX) [file pone.0186471.s001.docx]

|  | **Respondents reporting # of incidents^1^** | | **# of incidents reported by respondent** | | | | | | | |
| --- | --- | --- | --- | --- | --- | --- | --- | --- | --- | --- |
|  |  |  | **Sum total** | | **Mean** | | **Median** | **Q1** | **Q2** | **Std** |
| **Females (N=928)** | | |  | | | | | |  |  |
| Any type (N=261) | 250 | | 806 | | 3.2 | | 2 | 1 | 3 | 4.1 |
| Sexualized Touching (N=219) | 210 | | 432 | | 2.1 | | 1 | 1 | 2 | 2.4 |
| Penetrative Assault (N=126) | 115 | | 198 | | 1.7 | | 1 | 1 | 2 | 2.1 |
| Attempted penetrative Assault (N=103) | 98 | | 176 | | 1.8 | | 1 | 1 | 2 | 1.7 |
| **Males (N=634)** |  | |  |  | |  |  |  |  |  |
| Any type (N=79) | 77 | | 175 | | 2.3 | | 2 | 1 | 3 | 2.0 |
| Sexualized Touching (N=70) | 68 | | 105 | | 1.5 | | 1 | 1 | 1.5 | 1.1 |
| Penetrative Assault (N=33) | 33 | | 39 | | 1.2 | | 1 | 1 | 1 | 0.6 |
| Attempted penetrative Assault (N=24) | 22 | | 31 | | 1.4 | | 1 | 1 | 1 | 1.4 |
| **Other (N=26)** |  |  |  | |  | |  |  |  |  |
| Any type (N=10) | 9 | | 26 | | 2.9 | | 2 | 2 | 4 | 1.8 |
| Sexualized Touching (N=10) | 9 | | 21 | | 2.3 | | 2 | 1 | 3 | 1.6 |
| Penetrative assault (N=2) | - | | - | | - | | - | - | - | - |
| Attempted penetrative assault (N=2) | - | | - | | - | | - | - | - | - |

**S1 Table. Number of Incidents of Sexual Assault Since Enrolling at CU/BC, Among Individuals with At Least One Incident.**

^1^ N totals differ from number of respondents who reported ever experiencing sexual assault of that type since enrolling in college because of item non-response (some participants "refused to answer" the survey question asking about # of incidents, range=0%-1.1%) .
